# Orbital and Intracranial Complications of Acute Rhinosinusitis in a Tertiary Center, Saudi Arabia

**DOI:** 10.7759/cureus.42866

**Published:** 2023-08-02

**Authors:** Mohammed A Asiri, Mohammed H Almusallam, Yasser Almashari, Yazeed Allarakia, Riyadh A Alhedaithy

**Affiliations:** 1 Department of Otolaryngology, King Abdulaziz Medical City Riyadh, Riyadh, SAU; 2 College of Medicine, King Saud Bin Abdulaziz University for Health Sciences, Riyadh, SAU; 3 Division of Otolaryngology – Head and Neck Surgery, Department of Surgery, Ministry of National Guard - Health Affairs, Riyadh, SAU; 4 Division of Otolaryngology – Head and Neck Surgery, Department of Surgery, King Abdullah International Medical Research Center, Riyadh, SAU

**Keywords:** complication, rhinosinusitis, complications, intracranial, orbital

## Abstract

Background

Rhinosinusitis is an inflammatory condition affecting the nasal cavity’s mucosa and paranasal sinuses. In rare cases, acute rhinosinusitis (ARS) might lead to significant orbital and intracranial complications. This study aims to review the patients who presented with orbital or intracranial complications of ARS and to identify the main outcomes of these complications and their prognosis.

Methods

In this study, a retrospective chart review of patients with orbital or intracranial complications of ARS who presented to the otolaryngology department at King Abdulaziz Medical City, Riyadh, Saudi Arabia from 2016 to 2022 was conducted.

Results

A total of 43 patients with orbital, intracranial, or both (orbital and intracranial) complications of ARS were included. The most involved sinuses were maxillary sinuses. The most reported orbital complications were subperiosteal abscesses, and the most reported intracranial complications were epidural abscesses.

Conclusion

Orbital and intracranial complications of ARS are serious and life-threatening if not recognized early and treated effectively. The majority of ARS complications in this study were orbital complications. Fortunately, most of the cases carry a favorable outcome.

## Introduction

Rhinosinusitis is described as an inflammatory condition that affects the nasal cavity along with the paranasal sinuses [1]. The most common causative pathogens for acute rhinosinusitis (ARS) are viruses like rhinovirus, coronavirus, and adenovirus, along with bacterial and fungal organisms. It can affect children younger than 15 years and most commonly adults between 25 to 64 years. Moreover, it is one of the most common causes of clinical visits in the US [2]. Furthermore, acute rhinosinusitis is the fifth-most common reason for an antibiotic prescription (e.g., amoxicillin with or without clavulanate) accounting for one in five antibiotic prescriptions for adults, or a period of watchful waiting in some cases [3]. 

Complications of acute rhinosinusitis are rare, occurring in about 1 out of every 1000 cases, and are divided into orbital, intracranial, and bony complications [4]. The orbital spread of infection is the most common complication of acute rhinosinusitis due to the very thin ethmoid bone that separates infections from the ethmoid bone to the orbit [5]. According to Chandler's classification, orbital complications are divided based on their severity into preseptal cellulitis, orbital cellulitis, subperiosteal abscess, orbital abscess, and cavernous sinus thrombosis [6]. It is characterized by eyelid edema, erythema, chemosis, proptosis, blurred vision, fever, headache, and double vision [7]. Furthermore, intracranial complications, which are known to be complications of ARS, have potentially life-threatening consequences including subdural empyema, epidural and intracerebral abscess, meningitis, and sinus thrombosis [8-9]. In our study, we will focus on orbital and intracranial complications of acute rhinosinusitis to expand our perspective and identify the main outcomes and prognosis to shed light on their significance and effect among the patients.

## Materials and methods

King Abdullah International Medical Research Center (KAIMRC) issued approval for this study with an IRB No. of IRB/1756/22. After obtaining ethical approval, a retrospective cohort study was performed at King Abdulaziz Medical City in Riyadh, Saudi Arabia. Charts of all patients who visited the hospital and were diagnosed with orbital or intracranial complications of ARS from the period of January 2016 to December 2022 were reviewed. Data analysis was done using SPSS Statistics (IBM Corp., Armonk, NY). All statistical methods used were two-tailed with an alpha level of 0.05; if the p-value was less than or equal to 0.05, it was considered significant. Descriptive analysis was done by prescribing frequency distribution and percentage for study variables including patient's bio-demographic data, affected sinuses, clinical symptoms, and duration of symptoms. Cross tabulation to assess factors associated with orbital and cranial complications among ARS cases was carried out with Pearson chi-square test for significance and exact probability test if there were small frequency distributions.

## Results

A total of 43 patients with orbital, intracranial, or both (orbital and intracranial) complications of ARS were included. Patients' ages ranged from 1.5 to 69 years with a mean age of 16.2 ± 13.1 years old. Most of the included patients were males (Table 1).

**Table 1:** Demographic data of study patients with orbital or intracranial complications of ARS

Demographic data	No	%
Age in years		
< 10	13	30.2%
10-19	20	46.5%
20+	10	23.3%
Gender		
Male	25	58.1%
Female	18	41.9%

The most involved sinuses were maxillary sinuses. As for clinical presentation, eye swelling was the most reported clinical presentation. Symptoms lasted for one to three days for most of the hospitalized patients (Table 2).

**Table 2:** Clinical data of complicated ARS among study cases

Sinusitis clinical data	Count	Column N %
Affected sinus		
Maxillary	29	67.4%
Ethmoid	22	51.1%
Sphenoidal	13	30.2%
Frontal	11	25.5%
Presenting complaint		
Eye swelling	26	60.4%
Headache	17	39.5%
Fever	17	39.5%
Eye discharge	12	27.9%
Nasal discharge	4	9.3%
Seizures	3	7.0%
Vomiting	3	7.0%
Frontal swelling	1	2.3%
Ear discharge	1	2.3%
Duration of symptoms (days)		
1-3	21	48.8%
4-7	12	27.9%
8+	10	23.3%

**Figure 1:**
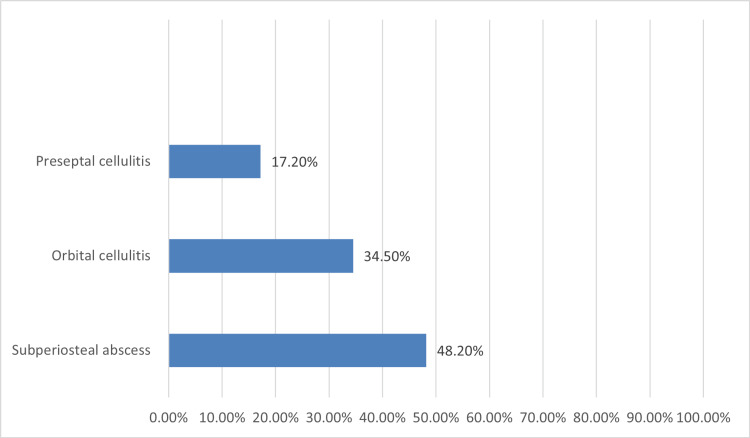
Orbital complications of ARS among study cases

A total of 29 cases had orbital complications with seven patients presenting with both orbital and intracranial complications. The most reported orbital complication was subperiosteal abscess (Figure 1).

**Figure 2:**
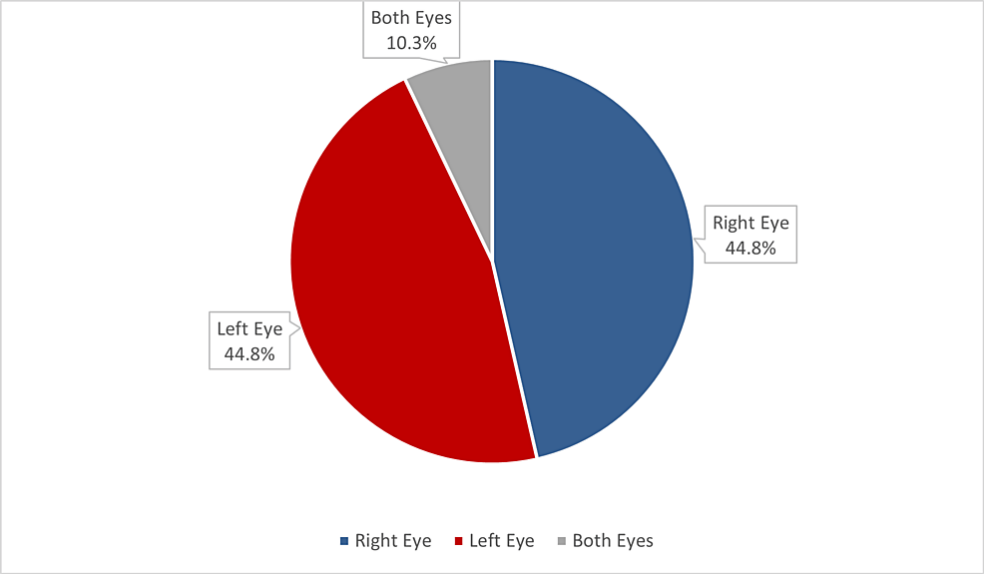
Side of the reported orbital complication of ARS among study cases

Thirteen of the study cases with orbital complications were affected in the right eye and the left eye equally (Figure 2). 

**Figure 3:**
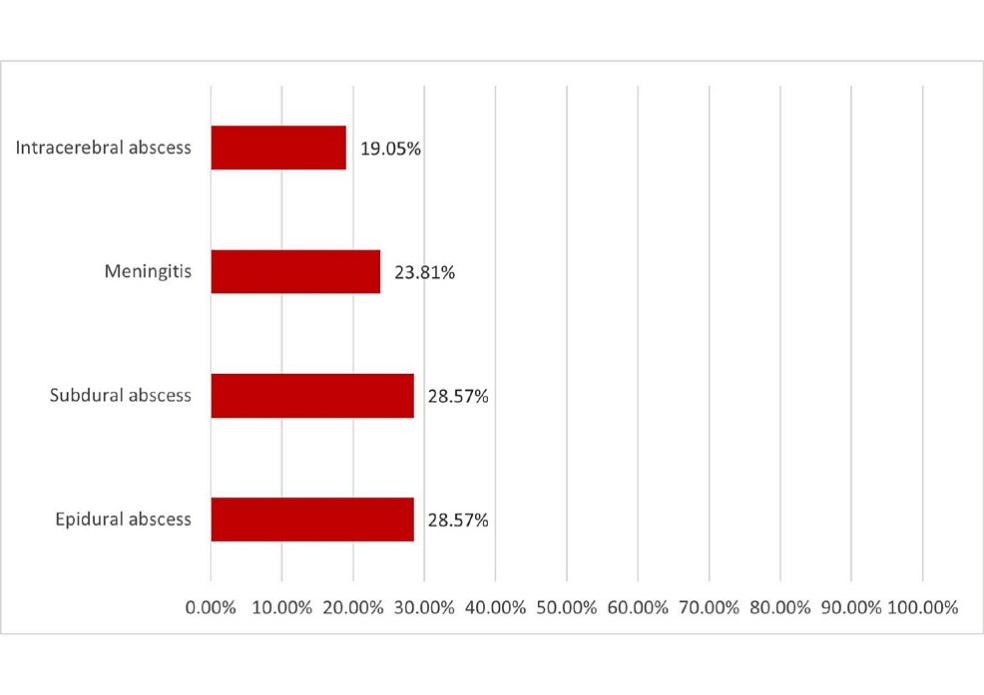
Intracranial complications of ARS among study cases

A total of 21 cases had intracranial complications due to ARS with seven patients presenting with both orbital and intracranial complications. The most reported complication was epidural abscess (Figure 3). 

A total of 22 of the study cases had orbital complications alone. Most of them were managed with antibiotics without surgical intervention. Around 14 of the cases had intracranial complications alone. Most of them were managed by antibiotics and surgical intervention. Seven cases had both orbital and intracranial complications, and all of them were managed by surgical intervention and antibiotics (Table 3).

**Table 3:** Clinical outcome of study patients with orbital or intracranial complications of ARS

Outcome	No	%
Management of patients with subperiosteal abscess alone		
Surgery and antibiotics	5	50%
Antibiotics only	5	50%
Management of patients with orbital cellulitis alone		
Surgery and antibiotics	1	12.5%
Antibiotics only	7	87.5%
Management of patients with preseptal cellulitis alone		
Surgery and antibiotics	0	0%
Antibiotics only	4	100%
Management of patients with epidural abscess alone		
Surgery and antibiotics	2	100%
Antibiotics only	0	0%
Management of patients with subdural abscess alone		
Surgery and antibiotics	3	100%
Antibiotics only	0	0%
Management of patients with intracerebral abscess alone		
Surgery and antibiotics	4	100%
Antibiotics only	0	0%
Management of patients with meningitis alone		
Surgery and antibiotics	1	20%
Antibiotics only	4	80%
Management of patients with subperiosteal abscess and epidural abscess		
Surgery and antibiotics	3	100%
Antibiotics only	0	0%
Management of patients with subperiosteal abscess and subdural abscess		
Surgery and antibiotics	1	100%
Antibiotics only	0	0%
Management of patients with orbital cellulitis and epidural abscess		
Surgery and antibiotics	1	100%
Antibiotics only	0	0%
Management of patients with orbital cellulitis and subdural abscess		
Surgery and antibiotics	1	100%
Antibiotics only	0	0%
Management of patients with preseptal cellulitis and subdural abscess		
Surgery and antibiotics	1	100%
Antibiotics only	0	0%
Length of stay		
< 1 week	16	37.2%
1-2 weeks	14	32.6%
> 2 weeks	13	30.2%
Prognosis		
Improved	41	95.3%
Death	1	2.3%
Recurrence of intracerebral abscess	1	2.3%

A total of six patients had surgical interventions for orbital complications. Around 10 patients had surgical interventions for intracranial complications, and seven patients had surgical interventions for both orbital and intracranial complications (Table 4).

**Table 4:** Surgical intervention of study patients with orbital, intracranial, or orbital and intracranial complications of ARS FESS: functional endoscopic sinus surgery

Surgical interventions	n	%
Surgical intervention in patients with orbital complications		
FESS only	2	33.30%
FESS + external drainage of orbital abscess	3	50%
Enucleation of eyeball with sphere implant	1	16.70%
Surgical intervention in patients with intracranial complications		
FESS only	1	10%
Craniotomy only	4	40%
FESS + craniotomy	3	30%
Burr hole only	1	10%
FESS + burr hole	1	10%
Surgical intervention in patients with orbital and intracranial complications		
FESS only	2	28.50%
FESS + craniotomy	2	28.50%
FESS + burr hole	1	14.30%
FESS + craniotomy + external drainage of subperiosteal abscess	2	28.50%

## Discussion

The most affected age group in our study is between 10 and 19 years in both orbital and intracranial complications. It has been found that patients who had symptoms for three days or less had orbital complications like orbital cellulitis, preseptal cellulitis, and subperiosteal abscess. On the other hand, the majority of intracranial complication cases presented to the emergency department eight days or more after having symptoms. This can indicate the time taken for the orbital complication to give rise to symptoms like eye swelling, eye discharge, fever, and other symptoms which is not as long as the time needed for intracranial complications to present with symptoms like headache, seizure, fever, nausea, and vomiting. In addition, many patients may present with multiple sinus involvement at first presentation which could indicate a more severe disease; thus, if not recognized early, and treated timely and effectively these cases might lead to unwanted orbital and intracranial complications.

In our study, it has been shown that the majority of ARS complications are orbital, which showed similarity to another study confirming the same result. According to Oxford et al. study with children who were admitted with complications of ARS, orbital cellulitis and orbital abscesses were the two most common complications accounting for 51 and 44 respectively [10]. Furthermore, complication like subperiosteal abscess was seen the most among the cases in our study with the most common presentation being eye swelling. As most of the patients had orbital complications, the majority of them presented to the emergency department with eye swelling. Headache was the second-most common presentation in all patients due to either the affected sinus itself, the orbital complication, or the extension to the cranial cavity.

Frontal and sphenoid sinus involvement in ARS is associated with a high risk of developing intracranial complications. According to the literature, the sphenoid sinus is considered one of the main sources of intracranial complications, which can be a result of its contiguous location to the cranial cavity [11]. Additionally, sphenoid sinus inflammation can present with hardly any rhinologic signs. Headache on the other hand can be the only presenting symptom, making it a challenging clinical diagnosis and requiring early CT imaging. Furthermore, the occurrence of seizures was noticed in intracranial complications and was mainly associated with subdural abscesses. In another study, it was shown that ethmoidal sinuses were the most affected location in their cases and the maxillary sinuses were third, while our study demonstrated the maxillary sinus to be the most affected. This can indicate the inconsistency in predicting which sinuses are involved and warrant radiological imaging like a CT scan or MRI to identify the affected sinus and manage it accordingly [12]. Sinus CT may show opacification, air-fluid levels, and inflammation. A thickened sinus mucosa over 5 mm is indicative of inflammation. It can also effectively assess bony erosion or destruction. MRI offers more detail than sinus CT to evaluate soft tissue or help elucidate tumors. Thus, MRI may be helpful to determine the extent of complications in cases such as an orbital or intracranial extension.

Orbital and intracranial complications can have similar presentations, such as fever due to inflammation or infection, or discharge, which can be orbital or nasal. These findings are also similar to another study that found headaches and fever in a major part of the study population [13]. Most of the patients had symptoms such as eye swelling, headache, and fever for less than three days before presenting to either the emergency department or the outpatient clinics. All patients were admitted, with a hospital stay of less than two weeks for most cases. However, a considerable number of patients were hospitalized for more than two weeks, indicating the long course of management for complicated ARS cases. Most of the patients were managed surgically in addition to antibiotics and symptomatic treatment, in which we found a favorable number of these cases had improved signs and symptoms. Surgical interventions included functional endoscopic sinus surgery to clear the involved sinuses and drain accessible subperiosteal abscesses, craniotomy or burr hole evacuation of brain abscesses, and evacuation of orbital abscesses through an external approach. Nonetheless, there has been a recurrence of abscess formation in one case, and one mortality was found among the patients.

This study has some limitations including the sample being taken from a single center hence, the variety in the variables is limited. Moreover, no follow-up updates were included regarding the patients after management, which would have established the efficacy of the management. Also, no comorbidities were included to determine whether their health status was compromised before the complications occurred. Finally, the majority of the sample were children and elderly patients, which can be a predisposing factor to developing orbital and intracranial complications, as their immunity might be compromised; thus, we recommend further studies to include more centers in order to include a larger population and a better view of such complications.

## Conclusions

Acute rhinosinusitis is a common disease with a rare possibility to cause complications. Orbital and intracranial complications of ARS are serious and life-threatening if not recognized early and treated effectively. The majority of ARS complications in this study were orbital complications with subperiosteal abscess being the most common type. Most patients with orbital complications have been treated conservatively with antibiotics. On the other hand, the majority of intracranial complications cases were managed surgically in addition to antibiotics.
